# Oral Frailty and Meal Positioning Associated With Aspiration Pneumonia in Long‐Term Care Residents at High Risk of Malnutrition

**DOI:** 10.1111/ggi.70757

**Published:** 2026-08-04

**Authors:** Xinze Wu, Tatsuma Okazaki, Jiro Okochi, Satoru Ebihara

**Affiliations:** ^1^ Department of Rehabilitation Medicine Tohoku University Graduate School of Medicine Sendai Japan; ^2^ Department of Health Development and Medicine Osaka University Graduate School of Medicine Osaka Japan; ^3^ Tatsumanosato Geriatric Health Services Facility Daito Japan

**Keywords:** aspiration pneumonia, meal positioning, modifiable care factors, oral health

## Abstract

**Background:**

Aspiration pneumonia (AP) remains a clinical problem in long‐term care facilities. However, evidence regarding modifiable care‐related factors influencing swallowing remains inconsistent. We used a nationwide survey of residents in Japanese long‐term care facilities to examine associations between oral frailty‐related conditions, meal positioning strategies, and AP.

**Methods:**

This cross‐sectional study analyzed nationwide survey data from residents at high risk of malnutrition in Japanese long‐term care facilities. AP status was determined based on facility medical records. Oral frailty‐related conditions and meal positioning factors were assessed using routine records. Logistic regression analyses evaluated associations with AP. Cumulative analysis examined associations between abnormal oral condition item counts and AP prevalence.

**Results:**

Among 1204 residents, 8.1% (*n* = 97) reported AP. After full adjustment, inability to gargle (OR = 1.991; 95% CI: 1.139–3.479) and unclear speech (OR = 1.752; 95% CI: 1.085–2.829) remained significantly associated with AP. During meals, reclining angles of 0°–30° (OR = 4.024; 95% CI: 1.006–16.106) and 30°–60° (OR = 1.890; 95% CI: 1.074–3.327) were associated with AP. AP prevalence increased from 2.9% in residents with 0–1 abnormal oral frailty‐related items to 13.1% in those with ≥ 4 items.

**Conclusion:**

AP was associated with cumulative oral frailty‐related conditions and meal positioning among residents at high risk of malnutrition in long‐term care facilities. Monitoring routine oral signs and optimizing seating angles may help reduce AP occurrence.

## Graphical abstract

Among nutritionally vulnerable long‐term care residents, aspiration pneumonia was associated with cumulative oral frailty and suboptimal feeding position, underscoring the importance of coordinated oral and feeding management in daily care.

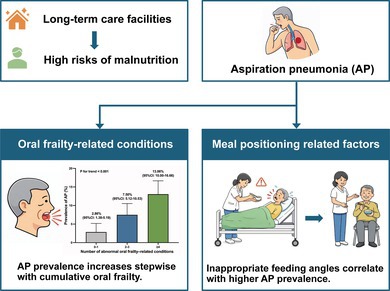

## 1 Introduction

Pneumonia remains a major public health concern and one of the leading causes of death among older adults [1]. Among its subtypes, aspiration pneumonia (AP) represents a major challenge in long‐term care settings [2, 3]. It is the leading cause of pneumonia among older adults with frailty receiving long‐term care and is associated with substantial morbidity and care burden [4, 5], particularly among residents who require daily assistance with feeding and oral care [6]. This burden may be greater in nutritionally vulnerable residents. Residents at high risk of malnutrition in long‐term care often have compromised oral function, reduced swallowing efficiency, and limited physiological reserve, which may increase susceptibility to AP [7, 8].

Previous studies have reported associations between oral frailty‐related conditions and AP [6, 9, 10], primarily through the colonization of the oral cavity by pathogenic microorganisms. Poor oral hygiene leads to colonization of the oral cavity by pathogenic microorganisms, which may serve as a reservoir for bacteria that can be aspirated into the lower respiratory tract [11]. Colonization of dental plaque by respiratory pathogens and subsequent aspiration of oropharyngeal bacteria have been consistently implicated in the pathogenesis of AP [9]. However, most existing studies have focused on oral frailty‐related conditions examined in isolation, often within limited clinical settings among residents with nutritional vulnerability [7, 12]. Evidence on the relationship of AP with the coexistence and overall burden of multiple oral frailty‐related conditions encountered in routine care environments in long‐term care populations remains limited.

In addition to oral frailty‐related conditions, feeding‐related factors have also been investigated in relation to AP [13]. Body position during meals may influence swallowing safety, with upright sitting generally associated with less difficulty than supine postures [14]. A semi‐recumbent position of approximately 30° may represent the optimal position for swallowing, as it facilitates bolus transit while maintaining airway protection and potentially reducing AP occurrence [15]. However, a study has reported that swallowing performance varies across positions, with subjective swallowing difficulty at 30° lower than that in the supine position but greater ease observed at more upright or 60° semi‐recumbent positions [16]. The observational findings in care settings regarding meal position and AP remain inconsistent [13]. These considerations may be relevant for long‐term care residents with greater functional and nutritional vulnerabilities in whom both oral function and postural control during feeding are often compromised. Given that meal‐related positioning is routinely adjusted in long‐term care facilities and is a potentially modifiable factor in daily care, further investigations are warranted.

Identifying potentially modifiable swallowing‐related factors in real‐world care environments is essential to develop practical strategies to reduce the occurrence of AP in long‐term care facilities. Therefore, this study aimed to investigate the associations among oral frailty‐related conditions, meal‐related positioning, and AP in a nationwide sample of residents with functional and nutritional vulnerability in Japanese long‐term care facilities.

## 2 Methods

### 2.1 Study Participants

**FIGURE 1:**
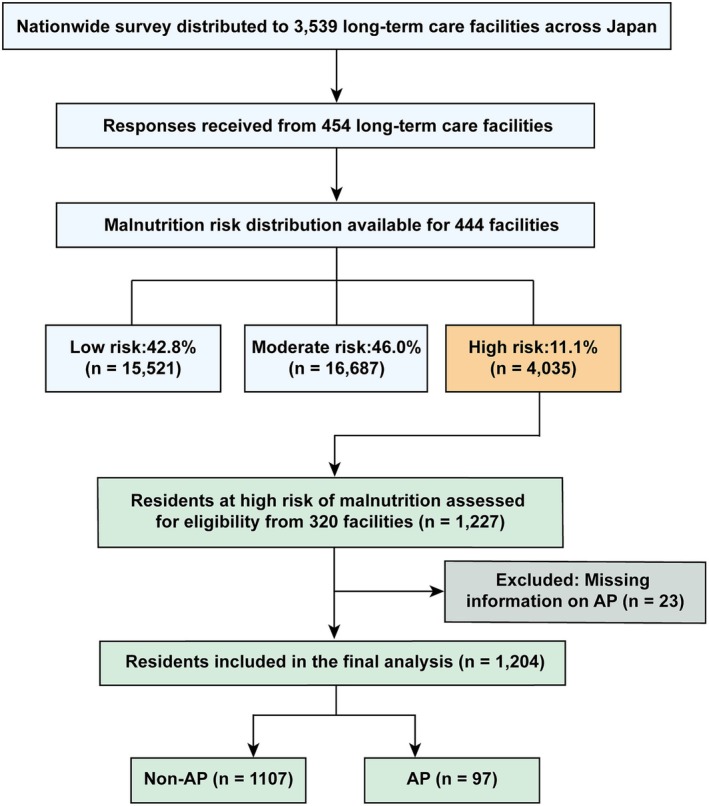
Flowchart of study participants. AP, aspiration pneumonia.

This study used individual‐level data derived from a nationwide survey conducted in Japanese long‐term care facilities (December 2024 to February 2025). The survey was implemented as part of a national project to investigate feeding and swallowing practices in long‐term care settings. Among the 454 facilities that responded to the survey, malnutrition risk distribution data were available for 444 facilities, which were used to characterize facility‐level nutrition risk. Based on these facility‐level data, 320 facilities reported the presence of residents classified as being at high risk of malnutrition and were therefore eligible for the individual‐level survey. For the individual‐level survey, each eligible facility was instructed to select up to five residents who met the predefined criteria for being at “high risk” of malnutrition as of the survey reference date (November 30, 2024); if fewer than five residents met these criteria, all eligible residents were included. Individual‐level data were collected only from residents who agreed to participate in the survey. Written informed consent was obtained, and the facility staff completed the survey forms based on routine daily care records. Accordingly, up to five residents per facility provided individual‐level data; a mean of 3.90 ± 1.45 residents provided data per facility. Of the 1227 residents surveyed, 23 were excluded because information on AP was missing, and 1204 residents were included for the final analysis (Figure 1). This study was approved by the institutional ethics committee.

### 2.2 Malnutrition Risk Classification

Malnutrition risk was classified according to the nutritional screening, assessment, and monitoring framework used in the Japanese long‐term care insurance system, rather than according to diagnostic criteria for malnutrition. This framework is routinely used in Japanese long‐term care facilities to stratify residents into low‐, moderate‐, and high‐risk categories based on nutritional and care‐related indicators.

Residents were classified as being at high risk of malnutrition if they met at least one of the following criteria: body mass index (BMI) < 18.5 kg/m^2^; weight loss of ≥ 5% within 1 month, ≥ 7.5% within 3 months, or ≥ 10% within 6 months; serum albumin concentration < 3.0 g/dL; food intake ≤ 75%; use of enteral or parenteral nutrition; or presence of pressure ulcers. Residents were classified as low risk only when all low‐risk criteria were satisfied, including BMI 18.5–29.9 kg/m^2^, no relevant weight loss, serum albumin concentration ≥ 3.6 g/dL, and food intake of 76%–100%, with no enteral or parenteral nutrition and no pressure ulcers. Residents who did not meet any high‐risk criteria but did not satisfy all low‐risk criteria were classified as moderate risk. This classification was used as an operational risk classification reflecting routine nutritional screening in Japanese long‐term care facilities. Although the Global Leadership Initiative on Malnutrition (GLIM) criteria are widely accepted for diagnosing malnutrition, GLIM‐based reassessment was not feasible in the present study because the dataset did not include all required GLIM components, particularly muscle mass and standardized information on disease burden or inflammation. For this reason, the present analysis used the Japanese long‐term care–based malnutrition risk classification to define the nutritionally at‐risk study population [17].

### 2.3 Assessment of AP

The AP status was determined based on the information documented in the facility medical records. Residents were classified according to whether an AP event had been recorded during the 3 months of observation preceding the survey reference date. Detailed clinical information was not consistently available across facilities; thus, AP was treated as a binary variable. AP was defined in accordance with the 2024 Japanese Respiratory Society Guidelines for Adult Pneumonia, which describe AP as “pneumonia occurring in individuals at risk of aspiration due to impaired swallowing function or gastroesophageal dysfunction.” Because detailed clinical information was not consistently available across facilities and there is no universally accepted diagnostic standard for AP [9], physician‐documented diagnoses in medical records were used, consistent with our previous studies. Pneumonia cases not reported as AP by the facility were not classified as AP in the present analysis.

### 2.4 Oral Frailty Related Assessment

Oral frailty‐related conditions were assessed using individual items routinely documented in care settings by facility staff involved in residents' daily care. The assessed oral frailty‐related items included difficulty opening the mouth, inability to firmly clench with bilateral posterior teeth, inability to gargle, halitosis, oral dryness, visible dental plaque, tongue coating, gingival swelling or bleeding, denture‐related problems, and unclear speech or conversation. Difficulty opening the mouth was defined as the inability to open the mouth sufficiently to accommodate approximately two fingerbreadths between the upper and lower anterior teeth. Inability to firmly clench bilateral posterior teeth was recorded when stable occlusion could not be achieved or when posterior teeth were absent despite denture use. Inability to gargle was defined as difficulty retaining water in the mouth or performing gargling during oral care. Halitosis and oral dryness were recorded when oral malodour or dryness was observed. Visible dental plaque and tongue coating were recorded based on observable deposits on tooth surfaces or the tongue. Gingival swelling or bleeding was recorded when gingival enlargement, redness, or bleeding was observed. Denture‐related problems were recorded when denture instability, poor fit, or related complaints were noted. Unclear speech or conversation was recorded when articulation or speech clarity was impaired.

### 2.5 Meal‐Related Positioning

Positioning during meals was assessed using individual items recorded in routine care records. This was based on daily observations by the facility staff during meals. The assessed items included the primary equipment used during meals (chair or bench, wheelchair, bed, or other), recline angle during meals (0°–30°, 30°–60°, > 60°). All variables were analyzed according to their original categorical classifications.

### 2.6 Covariates and Clinical Characteristics

Individual characteristics were obtained from routine care records. Clinical and demographic variables included age, sex, and BMI. Facilities type and care–need level were classified according to the Japanese Long‐Term Care Insurance system, with higher levels indicating greater functional dependency. Cognitive function was assessed using the Hasegawa Dementia Scale—Revised (HDS‐R) and Mini‐Mental State Examination (MMSE). Swallowing function was assessed using the five‐level ICF staging system for swallowing function, ranging from Stage 5, indicating the ability to chew and eat a regular diet including firm foods, to Stage 1, indicating no swallowing of dysphagia‐modified food [18]. The comorbidities recorded included neurological diseases, dementia, cardiovascular disease, hypertension, diabetes mellitus, other pulmonary diseases, and musculoskeletal disorders. Medication‐related characteristics included prescription medication use, number of prescribed medications, and the use of hypnotics, anti‐dementia drugs, and psychotropic drugs. These variables were recorded based on routine medication records and analyzed descriptively.

### 2.7 Statistical Analysis

Continuous variables were summarized as mean ± standard deviation (SD) and compared between groups using Student's *t*‐test or the Mann–Whitney *U* test, as appropriate. Categorical variables are summarized as counts and percentages and were compared using the chi‐square test or Fisher's exact test.

Logistic regression analyses were performed to evaluate the associations among oral frailty‐related conditions, meal positioning, and AP. Crude models were first developed. Model 1 was adjusted for age, sex, BMI, care–need level, and other pulmonary diseases. Model 2 was further adjusted for swallowing function assessed using the ICF swallowing staging system. Covariates were selected a priori based on clinical relevance and previous literature regarding risk factors for AP in older adults. The results were presented as odds ratios (ORs) with 95% confidence intervals (CIs). To evaluate the cumulative burden of oral frailty‐related conditions, a cumulative oral condition score was calculated as the total number of abnormal findings among the 10 predefined oral frailty‐related items, following the concept of deficit accumulation [19, 20]. Each abnormal item was coded as 1 and each normal item as 0, resulting in a total score ranging from 0 to 10. Based on the distribution of participants and AP cases, participants were categorized into three groups: 0–1, 2–3, and ≥ 4 abnormal items. AP prevalence and corresponding 95% CIs were calculated for each score category. Logistic regression analyses were performed to estimate ORs for AP across cumulative score categories. A trend test was conducted by treating the cumulative score category as an ordinal variable.

Statistical significance was defined as a two‐sided *p* < 0.05. The cumulative score analysis, prevalence estimates, 95% CIs, and trend test were performed using R software version 4.5.2 (R Foundation for Statistical Computing, Vienna, Austria). All other analyses were performed using SPSS version 29.0 (IBM Corp., Armonk, NY, USA).

## 3 Results

### 3.1 Baseline Characteristics

All residents included in the final analysis had high risks of malnutrition, and AP was reported in 8.1% (*n* = 97) based on facility records. Residents with AP had a significantly lower BMI (*p* = 0.011) and a lower proportion of females (*p* < 0.001) than those without AP. The care–need level differed significantly between the groups (*p* = 0.005), and Level 5 was assigned to a higher proportion of residents with AP. AP residents had significantly worse ICF swallowing stage distributions than non‐AP residents (*p* < 0.001), with higher proportions in Stages 3–5. No significant differences were observed in facility type distribution, cognitive function assessed using the HDS‐R or MMSE scores, or most comorbidities. However, other pulmonary diseases were significantly more prevalent among residents with AP (*p* < 0.001) (Table 1).

**TABLE 1:** Baseline characteristics of participants with and without AP.

Characteristics	Non‐AP (*n* = 1107)	AP (*n* = 97)	p
Age (years)	87.94 ± 7.99	87.93 ± 7.95	0.990
BMI (kg/m^2^)	18.89 ± 6.60	17.30 ± 2.92	0.011
Female, % (*n*)	73.3 (809)	56.3 (54)	< 0.001
Care–need level, % (*n*)			0.005
Level 1	6.6 (73)	2.1 (2)	
Level 2	11.4 (125)	6.3 (6)	
Level 3	21.9 (241)	21.9 (21)	
Level 4	35.5 (390)	29.2 (28)	
Level 5	24.5 (267)	40.6 (39)	
Facilities type, % (*n*)			0.100
Super‐enhanced type	46.3 (505)	51.1 (48)	
Enhanced type	13.4 (146)	10.6 (10)	
Add‐on type	24.8 (271)	24.5 (23)	
Basic type	14.3 (156)	11.7 (11)	
Other type	0.3 (3)	2.1 (2)	
Long‐term care medical type	0.9 (10)	0.0 (0)	
HDS‐R score	14.00 ± 7.97	10.18 ± 8.72	0.129
MMSE score	10.78 ± 7.89	8.83 ± 8.26	0.130
ICF swallowing stage, % (*n*)			< 0.001
1	24.2 (268)	5.2 (5)	
2	23.6 (261)	12.4 (12)	
3	14.9 (165)	20.6 (20)	
4	25.8 (286)	36.1 (35)	
5	4.7 (52)	16.5 (16)	
Chronic conditions, % (*n*)			
Neurological disease	11.2 (124)	9.3 (9)	0.735
Dementia	24.0 (266)	19.6 (19)	0.383
Cardiovascular disease	10.5 (116)	6.2 (6)	0.220
Hypertension	22.3 (247)	22.7 (22)	0.899
Diabetes mellitus	12.9 (143)	9.3 (9)	0.343
Pulmonary diseases	8.3 (92)	23.7 (23)	< 0.001
Musculoskeletal disorders	17.4 (193)	14.4 (14)	0.574
Medication use			
Prescription medication use	96.5 (1047)	92.7 (89)	0.086
Number of prescribed medications	5.53 ± 2.95	5.28 ± 2.47	0.456
Use of hypnotics	24.9 (276)	18.6 (18)	0.224
Use of anti‐dementia drugs	11.9 (132)	10.3 (10)	0.861
Use of psychotropic drugs	23.1 (256)	21.6 (21)	0.924

Abbreviations: AP, aspiration pneumonia; BMI, body mass index; HDS‐R, Hasegawa Dementia Scale—Revised; MMSE, Mini Mental State Examination.

### 3.2 Oral Frailty‐Related Conditions Associated With AP

Residents with AP more frequently reported significantly poorer oral functional status, including the inability to clench bilateral posterior teeth (*p* = 0.002), inability to gargle (*p* < 0.001), presence of halitosis (*p* = 0.016), oral dryness (*p* = 0.005), tongue coating (*p* = 0.018), gingival swelling or bleeding (*p* = 0.048), and unclear speech or conversation (*p* < 0.001) (Table S1).

### 3.3 Meal‐Related Positioning and Care Practices Associated With AP

Significant differences were observed in the primary seating equipment used during meals. Residents with AP were more likely to eat meals in bed and less likely to use chairs or benches (*p* < 0.001). The recline angle during meals also differed significantly, with residents with AP consuming meals more frequently at lower reclining angles (*p* < 0.001) (Table S2).

### 3.4 Associations Between Oral Frailty‐Related Condition and AP

Logistic regression analyses were performed to examine the association between oral frailty‐related conditions and AP. The crude analyses showed that inability to clench with bilateral posterior teeth (OR = 1.952; 95% CI: 1.275–2.991; *p* = 0.002), inability to gargle (OR = 3.670; 95% CI: 2.361–5.703; *p* < 0.001), halitosis (OR = 1.855; 95% CI: 1.152–2.988; *p* = 0.011), oral dryness (OR = 1.981; 95% CI: 1.259–3.117; *p* = 0.003), tongue coating (OR = 1.673; 95% CI: 1.100–2.546; *p* = 0.016), gingival swelling or bleeding (OR = 1.666; 95% CI: 1.023–2.713; *p* = 0.040), and unclear speech (OR = 2.881; 95% CI: 1.875–4.428; *p* < 0.001) were significantly associated with AP. After adjustment, the inability to gargle (OR = 2.983; 95% CI: 1.789–4.976; *p* < 0.001) and unclear speech (OR = 2.037; 95% CI: 1.276–3.251; *p* = 0.003) remained significantly associated with AP. And halitosis (OR = 1.727; 95% CI: 1.029–2.897; *p* = 0.039) also showed significant associations after adjustment (Table 2). We further adjusted for swallowing function status assessed using the ICF staging system. In this model, inability to gargle (OR = 1.991; 95% CI: 1.139–3.479; *p* = 0.016) and unclear speech (OR = 1.752; 95% CI: 1.085–2.829; *p* = 0.022) remained significantly associated with AP (Table 2).

**TABLE 2:** Associations between oral frailty‐related conditions and AP.

Variables	Crude		Model 1		Model 2	
	OR (95% CI)	*p*	OR (95% CI)	*p*	OR (95% CI)	*p*
Difficulty opening the mouth						
Present	1.426 (0.691–2.946)	0.337	1.376 (0.635–2.978)	0.418	1.211 (0.550–2.665)	0.635
Absent	Reference	/	Reference	/	Reference	/
Inability to firmly clench with bilateral posterior teeth						
Unable	1.952 (1.275–2.991)	0.002	1.344 (0.841–2.146)	0.216	1.028 (0.624–1.692)	0.915
Able	Reference	/	Reference	/	Reference	/
Inability to gargle						
Unable	3.670 (2.361–5.703)	< 0.001	2.983 (1.789–4.976)	< 0.001	1.991 (1.139–3.479)	0.016
Able	Reference	/	Reference	/	Reference	/
Halitosis						
Present	1.855 (1.152–2.988)	0.011	1.727 (1.029–2.897)	0.039	1.655 (0.980–2.794)	0.060
Absent	Reference	/	Reference	/	Reference	/
Oral dryness						
Present	1.981 (1.259–3.117)	0.003	1.615 (0.99–2.631)	0.054	1.425 (0.862–2.357)	0.167
Absent	Reference	/	Reference	/	Reference	/
Visible dental plaque						
Present	1.320 (0.867–2.012)	0.196	1.405 (0.894–2.208)	0.141	1.309 (0.833–2.058)	0.244
Absent	Reference	/	Reference	/	Reference	/
Tongue coating						
Present	1.673 (1.100–2.546)	0.016	1.445 (0.924–2.259)	0.107	1.396 (0.888–2.196)	0.148
Absent	Reference	/	Reference	/	Reference	/
Gingival swelling or bleeding						
Present	1.666 (1.023–2.713)	0.040	1.462 (0.863–2.477)	0.157	1.316 (0.772–2.241)	0.313
Absent	Reference	/	Reference	/	Reference	/
Denture‐related problems						
Present	1.342 (0.763–2.360)	0.308	1.435 (0.794–2.591)	0.231	1.412 (0.778–2.561)	0.256
Absent	Reference	/	Reference	/	Reference	/
Unclear speech or conversation						
Present	2.881 (1.875–4.428)	< 0.001	2.037 (1.276–3.251)	0.003	1.752 (1.085–2.829)	0.022
Absent	Reference	/	Reference	/	Reference	/

*Note:* Model 1: Adjusted for age, sex, BMI, care–need level, and lung disease. Model 2: further adjusted for swallowing function status assessed using the ICF stage.

Abbreviation: AP, aspiration pneumonia.

### 3.5 Associations Between Meal‐Related Positioning and AP

In the crude analyses, eating meals in bed (OR = 5.082; 95% CI: 2.042–12.645; *p* < 0.001) and using other non‐standard seating equipment (OR = 23.714; 95% CI: 2.902–193.811; *p* = 0.003) were significantly associated with AP. Lower reclining angles during meals were also associated with increased odds of AP, with significantly higher risks observed at reclining angles of 0°–30° (OR = 5.043; 95% CI: 1.343–18.940; *p* = 0.017) and 30°–60° (OR = 2.884; 95% CI: 1.764–4.716; *p* < 0.001) than at angles > 60°. The association between lower reclining angles and AP persisted in the adjusted model. Compared with recline angles > 60°, both 0°–30° (OR = 5.666; 95% CI: 1.424–22.540; *p* = 0.013) and 30°–60° (OR = 2.253; 95% CI: 1.307–3.887; *p* = 0.004) were associated with increased odds of AP (Table 3). We further adjusted for swallowing function, reclining angles of 0°–30° (OR = 4.024; 95% CI: 1.006–16.106; *p* = 0.049) and 30°–60° (OR = 1.890; 95% CI: 1.074–3.327; *p* = 0.027) were also significantly associated with higher odds of AP compared with angles > 60° (Table 3).

**TABLE 3:** Associations between meal positioning and AP.

Variables	Crude		Model 1		Model 2	
	OR (95% CI)	*p*	OR (95% CI)	*p*	OR (95% CI)	*p*
Primary seating equipment during meals						
Chair or bench	Reference	/	Reference	/	Reference	/
Wheelchair	1.700 (0.763–3.791)	0.195	1.144 (0.484–2.703)	0.759	0.977 (0.406–2.353)	0.959
Bed	5.082 (2.042–12.645)	< 0.001	2.590 (0.938–7.150)	0.066	1.781 (0.625–5.071)	0.280
The other	23.714 (2.902–193.811)	0.003	15.390 (1.625–145.773)	0.017	16.254 (1.466–180.172)	0.023
Recline angle during meals						
0°–30°	5.043 (1.343–18.940)	0.017	5.666 (1.424–22.540)	0.013	4.024 (1.006–16.106)	0.049
30°–60°	2.884 (1.764–4.716)	< 0.001	2.253 (1.307–3.887)	0.003	1.890 (1.074–3.327)	0.027
> 60°	Reference	/	Reference	/	Reference	/

*Note:* Model 1: adjusted for age, sex, BMI, care–need level, and lung disease. Model 2: further adjusted for swallowing function status assessed using the ICF stage.

Abbreviation: AP, aspiration pneumonia.

### 3.6 Cumulative Abnormal Oral Frailty‐Related Items

The cumulative number of oral frailty‐related conditions was calculated based on all 10 predefined oral frailty‐related items (Table 4). Based on the distribution of participants and AP cases, participants were categorized into three groups according to the number of abnormal items: 0–1, 2–3, and ≥ 4. The prevalence of AP increased progressively across these categories, from 2.9% among residents with 0–1 abnormal item to 7.5% among those with 2–3 abnormal items and 13.1% among those with ≥ 4 abnormal items. In logistic regression, compared with residents with 0–1 abnormal items, those with 2–3 abnormal items had higher odds of AP (OR = 2.757; 95% CI: 1.373–6.015; *p* = 0.007), and those with ≥ 4 abnormal items had further increased odds of AP (OR = 5.109; 95% CI: 2.677–10.792; *p* < 0.001). A significant increasing trend was observed across cumulative oral condition score categories (*p* for trend < 0.001).

**TABLE 4:** Association between the cumulative number of oral frailty‐related conditions and AP.

Cumulative number of oral frailty‐related conditions	Participants, *n*	AP, *n* (%)	OR (95% CI)	*p*
0–1	350	10 (2.9)	Reference	/
2–3	400	30 (7.5)	2.757 (1.373–6.015)	0.007
≥ 4	421	55 (13.1)	5.109 (2.677–10.792)	< 0.001
*p* for trend				< 0.001

*Note:* The cumulative number of oral frailty‐related conditions was determined by counting each oral condition present. Participants were categorized into three groups according to the total number of abnormal items: 0–1, 2–3, and ≥ 4.

Abbreviation: AP, aspiration pneumonia.

## 4 Discussion

In this study, AP was associated with the cumulative burden of routinely observed oral frailty‐related conditions among long‐term care residents at nutritional risk. Rather than being linked to a single oral abnormality, AP prevalence increased progressively with the accumulation of multiple oral frailty‐related conditions. These findings suggest that oral frailty may be more appropriately viewed as a multidimensional clinical construct reflecting overall oral vulnerability rather than isolated impairments. In addition, meal positioning during feeding was also associated with AP. Our findings support a comprehensive approach that considers both oral frailty burden and feeding‐related care when assessing AP risk in long‐term care settings.

The present study demonstrated a graded association between the prevalence of AP and the cumulative number of abnormal oral frailty‐related condition items derived from routinely recorded care data. This finding extends previous research by showing that coexisting oral frailty‐related conditions are associated with higher AP prevalence than any single condition. While previous studies have reported associations between individual oral conditions and AP [6, 21, 22], these have largely been examined in isolation. Our results underscore the clinical relevance of considering oral frailty‐related conditions as a cumulative burden that closely reflects the complex oral functional impairments encountered in routine long‐term care. The cumulative oral frailty‐related condition score was constructed from 10 routinely assessed oral frailty‐related items and demonstrated a clear dose–response relationship with AP. This finding suggests that AP risk may be more closely related to the overall burden of oral functional impairment than to any single oral abnormality. Oral frailty is a multidimensional condition encompassing impairments in oral hygiene, oral motor function, swallowing‐related behaviors, and communication. Evaluating oral frailty‐related conditions as a cumulative burden may better reflect the complex oral health status of long‐term care residents than focusing on individual abnormalities alone.

These conditions may reflect a reduced oral self‐cleansing capacity, increased oral bacterial burden, and impaired coordination of oropharyngeal and laryngeal functions. A pilot study combining oral assessment with microbiological evaluation demonstrated that elevated oral bacterial levels are associated with a higher prevalence of AP, supporting the oral cavity as an important reservoir of pathogenic microorganisms [23]. Impaired oral self‐care, as indicated by the inability to gargle, may facilitate the accumulation of pathogenic microorganisms and increase the AP risk. Likewise, unclear speech and breathing discomfort may indicate compromised neuromuscular coordination between swallowing and respiration, which is essential for effective airway protection [24, 25]. Emerging evidence suggests that respiratory and swallowing functions are functionally linked and potentially modifiable. For example, respiratory muscle training has been associated with improved swallowing performance [26], and respiratory rehabilitation studies further indicate that breathing–swallowing coordination may be modifiable [26, 27]. Although such interventions were not examined in this study, these findings support that breathing‐related symptoms observed during meals may represent potentially modifiable targets.

In addition to oral factors, meal positioning was significantly associated with AP in this study. Residents who were fed at smaller reclining angles were more likely to have AP, whereas residents without AP more frequently fed at steeper angles, particularly those exceeding 60°. This finding is consistent with previous evidence demonstrating that AP is more common among bedridden older adults who remain in a supine position, and aspiration of gastric contents is less likely to occur in a semi‐recumbent position [28]. Prior studies have shown that maintaining 30° or more reduces the incidence of pneumonia, including hospital‐acquired pneumonia, and postprandial positioning at angles of 30° or higher for at least 2 h can significantly reduce febrile episodes among institutionalized older individuals [28, 29]. Semi‐recumbent positioning reduces the risk of AP in patients receiving enteral feeding [30]. Our results extend this evidence by demonstrating an association between the feeding angle and AP prevalence.

Meal positioning was also associated with AP in the present study. However, this finding should be interpreted with caution because meal positioning in long‐term care settings is often determined by healthcare professionals or caregivers according to an individual resident's swallowing ability and perceived AP risk. The observed association may partly reflect clinical decision‐making and compensatory care strategies rather than a direct causal effect of positioning itself. Nevertheless, the association remained after adjustment for swallowing function and may still provide clinically relevant information. One possible interpretation is that residents requiring lower reclining angles represent a population with greater underlying vulnerability to aspiration. In such individuals, positioning adjustments may be implemented as a risk‐reduction strategy, yet AP may still occur despite these interventions. Rather than acting through a single mechanism, feeding positioning may influence the prevalence of AP through multiple physiological pathways related to respiratory mechanics and swallowing function [31, 32]. A previous study reported that inspiratory force, expiratory force, and cough peak expiratory flow reached their maximum values at a trunk angle of 60° [33]. Because reduced respiratory muscle strength is associated with impaired cough effectiveness and increased AP risk in older adults [34], steeper angles may facilitate more effective clearance [35]; this may partly explain why residents who fed at angles exceeding 60° had a lower prevalence of AP in this study. Beyond respiratory mechanics, postural alignment influences the bolus flow and pharyngeal clearance during swallowing. Inadequate trunk elevation may increase pharyngeal residue and prolong bolus retention. This increases the risk of post‐swallowing AP, particularly in individuals with delayed swallowing initiation or reduced sensory feedback [36]. Feeding positioning is a modifiable bedside care practice that requires no specialized equipment and may represent a practical care target for nutritionally vulnerable residents in long‐term care settings.

An important contextual feature of this study is the focus on residents at high risk of malnutrition. In this population, nutritional vulnerability provides a shared clinical background, in which both oral frailty‐related conditions and feeding‐related factors are commonly observed during daily care. Malnutrition is associated with reduced muscle strength, diminished physiological reserve, and overall functional vulnerability [37], which may predispose residents to impaired oral function and difficulty maintaining an appropriate feeding posture. In this care context, oral conditions and feeding positioning should not be viewed as isolated or competing associated factors for AP but rather as complementary domains that are routinely addressed together during assistance in long‐term care settings. Residents with impaired oral function may have reduced physiological reserve for airway protection [38], and therefore require greater attention to feeding positioning during meals. From a practical care perspective, these factors represent different but complementary targets within the same feeding process rather than interdependent mechanisms. Our findings underscore the importance of integrated care approaches that address both oral function and feeding positioning among long‐term care residents at high risk of malnutrition [28].

### 4.1 Limitation

Despite the use of nationwide data from Japanese long‐term care facilities, this study has several important limitations that should be considered. First, the cross‐sectional design precludes causal inference. AP was identified based on events occurring during the preceding 3 months, whereas oral frailty‐related conditions and feeding posture were assessed from routine care records. Therefore, the temporal sequence between these factors and AP could not be determined. AP itself may contribute to subsequent functional decline, worsening oral conditions, and changes in feeding posture. In addition, feeding posture may have been modified by caregivers according to residents' swallowing ability and perceived aspiration risk. Accordingly, the observed associations should be interpreted as correlates of AP rather than evidence of causality.

Second, AP was identified based on facility‐reported events recorded in routine medical and care records rather than independent adjudication using a standardized diagnostic protocol. Although we adopted a broad operational definition of AP consistent with Japanese clinical guidelines, diagnostic practices may have varied across facilities. In addition, pneumonia cases not reported as AP were not classified as AP in the present analysis, and outcome misclassification cannot be completely excluded.

Third, oral conditions and meal‐related positioning were based on routinely documented care items rather than validated assessment instruments. Information on assessor training, calibration procedures, and inter‐rater reliability was unavailable. Detailed dental information, such as the number of remaining teeth and toothbrushing habits, was unavailable, and only trunk angle during feeding was recorded, whereas head and neck positioning were not assessed. Furthermore, because data were collected from multiple long‐term care facilities, differences in care practices, staffing, feeding support, and diagnostic procedures may have introduced facility‐level heterogeneity. Although a sensitivity analysis accounting for facility clustering produced generally similar findings, residual facility‐level confounding cannot be excluded. Other potentially relevant medical factors, including the severity of pulmonary disease, detailed neurological disease status, gastroesophageal reflux, and the duration or severity of dysphagia, were not captured in the dataset; residual medical confounding cannot be excluded.

Finally, the study population was restricted to residents classified as being at high risk of malnutrition using the Japanese long‐term care insurance nutritional risk classification, rather than standardized diagnostic criteria. Future studies are needed to validate these findings in more diverse long‐term care populations beyond residents at high risk of malnutrition.

## 5 Conclusion

Our findings indicate that the cumulative presence of adverse oral conditions is associated with higher AP prevalence. Feeding positioning, particularly the reclining angle during meals, is also significantly associated with AP. These factors reflect modifiable aspects of daily care practice. Targeted approaches focusing on comprehensive oral‐related management and careful feeding positioning may therefore represent practical strategies for addressing AP in institutionalized older populations.

## Author Contributions

S.E. conceived and supervised the study. X.W. performed the statistical analyses and drafted the manuscript. T.O. critically reviewed and revised the manuscript. J.O. provided the dataset and contributed to data verification and manuscript review. All authors reviewed and approved the final manuscript.

## Funding

This work was supported by the Japan Society for the Promotion of Science (JSPS) KAKENHI (22K19760, 24K02778, and 25K22899), Japan Agency for Medical Research and Development (25zf0127001h0005), JST Strategic International Collaborative Research Program (SICORP) (JPMJSC2308), Research Funding for Longevity Sciences from the National Center for Geriatrics and Gerontology (25‐29), and Grant from the Japan Association of Geriatric Health Service Facilities (Dysphagia Statement Review Project).

## Conflicts of Interest

The authors declare no conflicts of interest.

## Supporting information


**Table S1:** Comparison of oral conditions of residents with and without AP.
**Table S2:** Meal positioning‐related characteristics of residents with and without AP.

## Data Availability

The data underlying this article cannot be shared publicly due to restrictions imposed by the data‐providing organization. Data are available from the corresponding author upon reasonable request and with permission from the data provider.
