# Supplementary material for: Oral Frailty and Meal Positioning Associated With Aspiration Pneumonia in Long‐Term Care Residents at High Risk of Malnutrition
Source: Geriatr Gerontol Int. 2026 Aug 4;26(8):e70757. doi: 10.1111/ggi.70757 (PMC13434364; doi:10.1111/ggi.70757)
Supplement: Supplementary file 1 [file GGI-26-0-s001.docx]

Supplementary Table S1. Comparison of oral conditions of residents with and without AP

| Variable | Non-AP (n=1107) | AP (n=97) | P |
| --- | --- | --- | --- |
| Difficulty opening the mouth, % (n) | | | 0.301 |
| Able | 93.3 (1032) | 90.7 (88) |  |
| Unable | 6.7 (74) | 9.3 (9) |  |
| Inability to firmly clench with bilateral posterior teeth, % (n) | | | 0.002 |
| Able | 54.6 (602) | 38.1 (37) |  |
| Unable | 45.4 (500) | 61.9 (60) |  |
| Inability to gargle, % (n) |  |  | <0.001 |
| Able | 64.4 (710) | 33.0 (32) |  |
| Unable | 35.6 (393) | 67.0 (65) |  |
| Halitosis(%/n) |  |  | 0.016 |
| Present | 16.5 (182) | 26.8 (26) |  |
| Absent | 83.5 (922) | 73.2 (71) |  |
| Oral dryness, % (n) |  |  | 0.005 |
| Present | 19.4 (214) | 32.3 (31) |  |
| Absent | 80.6 (889) | 67.7 (65) |  |
| Visible dental plaque, % (n) |  |  | 0.205 |
| Present | 48.1 (530) | 41.2 (40) |  |
| Absent | 51.9 (572) | 58.8 (57) |  |
| Tongue coating, % (n) |  |  | 0.018 |
| Present | 42.4 (467) | 55.2 (53) |  |
| Absent | 57.6 (634) | 44.8 (43) |  |
| Gingival swelling or bleeding, % (n) |  |  | 0.048 |
| Present | 16.5 (182) | 24.7 (24) |  |
| Absent | 83.5 (922) | 75.3 (73) |  |
| Denture-related problems, % (n) |  |  | 0.345 |
| Present | 12.8 (140) | 16.5 (16) |  |
| Absent | 87.2 (951) | 83.5 (81) |  |
| Unclear speech or conversation, % (n) |  |  | <0.001 |
| Present | 37.0 (407) | 62.9 (61) |  |
| Absent | 63.0 (692) | 37.1 (36) |  |

AP: aspiration pneumonia

Supplementary Table S2. Meal positioning-related characteristics of residents with and without AP

| Variable | Non-AP (n=1107) | AP (n=97) | P |
| --- | --- | --- | --- |
| Primary seating equipment during meals (%/n) | |  | <0.001 |
| Chair or bench | 15.6 (166) | 8.2 (7) |  |
| Wheelchair | 76.2 (809) | 68.2 (58) |  |
| Bed | 7.9 (84) | 21.2 (18) |  |
| The other | 0.2 (2) | 2.4 (2) |  |
| Recline angle during meals (%/n) | |  | <0.001 |
| 0–30° | 1.0 (10) | 3.8 (3) |  |
| 30–60° | 17.4 (169) | 36.7 (29) |  |
| >60° | 81.5 (790) | 59.5 (47) |  |

AP: aspiration pneumonia
